# Mechanisims of asthma and allergic disease - 1088. Increased serum strail level in newly diagnosed stave-iv lung adenocarcinoma but not sqamous cell carcinoma, is correlated with age and smoking

**DOI:** 10.1186/1939-4551-6-S1-P84

**Published:** 2013-04-23

**Authors:** Aysegul Kargi, Atil Bisgin, Arzu Didem Yalcin, Bulent Kargi, Emel Sahin, Saadet Gumuslu

**Affiliations:** 1 Department of Internal Medicine, Antalya Education and Research Hospital, Antalya, Turkey; 2 Cancer Institute, Sweden; 3 Internal Medicine, Allergy and Immunology, Education and Research Hospital, Turkey; 4 Medical Park Hospital, Turkey; 5 Akdeniz University, Turkey

## Background

Clinical significance of serum sTRAIL and 1,25-dihydroxyvitamin D(3) level in patients with non-small cell lung cancer was investigated.

## Methods

Consecutive 18 adenocarcinoma and 22 squamous cell carcinoma patients of non-small cell lung cancer referred to our institute were included in this study. There were 12 men and 6 women, ages ranged from 38 to 97 years, with adenocarcinoma and average of 60.5 years. And there were 20 men and 2 women, ages ranged from 46 to 80 years, with squamous cell carcinoma and average of 64.95 years. Curative resection was performed for all patients. Serum levels of sTRAIL and 1,25-dihydroxyvitamin D(3) were measured in the samples of time of diagnosis.

## Results

Circulating sTRAIL levels of NSCLC patients were significantly higher than the control group. Although there was no correlation between patient survival and sTRAIL levels, the high sTRAIL levels in adenocarcinoma patients were correlated with age and cigarette smoking. Interestingly, the sTRAIL level of healthy individuals was correlated with serum 1,25-dihydroxyvitamin D(3).

## Conclusions

Serum sTRAIL concentrations changed significantly during NSCLC perpetuation and correlated with age and smoking in adenocarcinoma. However, it seems that the concentration of this protein has no critical value as a prognostic factor and no effect on survival rate in NSCLC.

